# SeisMote: A Multi-Sensor Wireless Platform for Cardiovascular Monitoring in Laboratory, Daily Life, and Telemedicine

**DOI:** 10.3390/s20030680

**Published:** 2020-01-26

**Authors:** Marco Di Rienzo, Giovannibattista Rizzo, Zeynep Melike Işilay, Prospero Lombardi

**Affiliations:** IRCCS Fondazione Don Carlo Gnocchi, 20148 Milano, Italy; frizzo@dongnocchi.it (G.R.); zisilay@dongnocchi.it (Z.M.I.); plombardi@dongnocchi.it (P.L.)

**Keywords:** body sensor network, wearable sensor, telemedicine, telerehabilitation, seismocardiogram, acceleration, electrocardiogram, cardiac mechanics, photoplethysmogram, pulse transit time

## Abstract

This article presents a new wearable platform, SeisMote, for the monitoring of cardiovascular function in controlled conditions and daily life. It consists of a wireless network of sensorized nodes providing simultaneous multiple measures of electrocardiogram (ECG), acceleration, rotational velocity, and photoplethysmogram (PPG) from different body areas. A custom low-power transmission protocol was developed to allow the concomitant real-time monitoring of 32 signals (16 bit @200 Hz) from up to 12 nodes with a jitter in the among-node time synchronization lower than 0.2 ms. The BluetoothLE protocol may be used when only a single node is needed. Data can also be collected in the off-line mode. Seismocardiogram and pulse transit times can be derived from the collected data to obtain additional information on cardiac mechanics and vascular characteristics. The employment of the system in the field showed recordings without data gaps caused by transmission errors, and the duration of each battery charge exceeded 16 h. The system is currently used to investigate strategies of hemodynamic regulation in different vascular districts (through a multisite assessment of ECG and PPG) and to study the propagation of precordial vibrations along the thorax. The single-node version is presently exploited to monitor cardiac patients during telerehabilitation.

## 1. Introduction

Over the years, there has been a growing demand for wearable systems able to monitor the cardiovascular function out of laboratory settings in ambulant subjects. The electrocardiogram (ECG) was the first signal to be monitored by this class of devices since the early 1960s (ECG Holter monitors) [1]. More recently, additional signals have also been considered for the evaluation of cardiac function in daily life. One of them is the seismocardiogram (SCG); this is the measure of minute thorax accelerations produced by the beating heart and can be simply detected by placing an accelerometer on the chest surface [2]. Usually, only the dorso-ventral component of the acceleration (corresponding to the *z*-axis of our sensor) is considered for SCG measurement. From the analysis of this signal, it is possible to obtain information on different mechanical events of the cardiac cycle including opening and closing of the aortic and mitral valves, atrial systole, and isovolumic contraction and relaxation [3,4,5,6].

Traditionally, cardiac mechanics are evaluated by ultrasound (US) techniques. This methodology offers a detailed investigation of heart performance, but it cannot be exploited for obtaining measurements outside of laboratory settings, and it cannot be used for studying the dynamic features of cardiac mechanics over time. This is because it provides snapshot measurements and also because of the considerable size of the device and the complexity of the assessments requiring expert operators. Conversely, monitoring by SCG allows repeated estimations of mechanical cardiac indexes either in controlled conditions and during outdoor living or at home through wearable devices that can be easily self-managed [7]. Because of all these features, the use of an SCG signal opens new opportunities for the investigation of cardiac mechanics in research and clinics [8,9,10,11].

**Figure 1:**
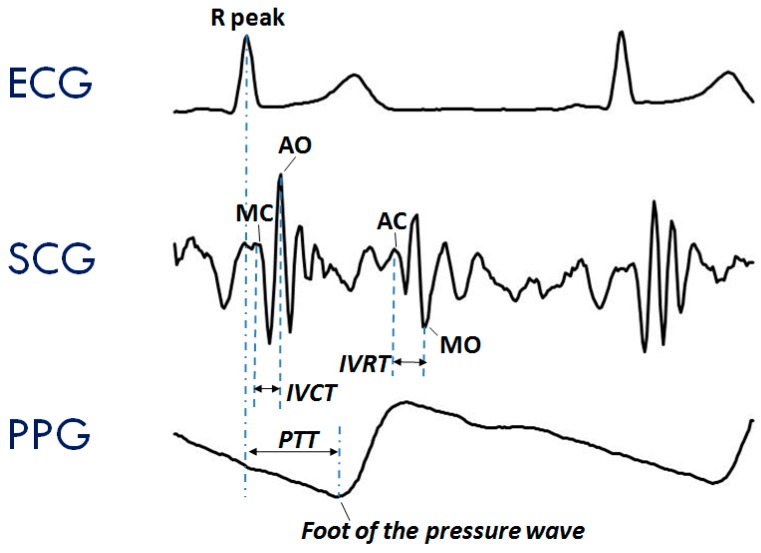
Typical waveforms of the seismocardiogram (SCG) and photoplethysmogram (PPG) as compared with the electrocardiogram (ECG). (**Upper Panel**) ECG signal with indication of the *Rpeak* fiducial point used for the estimation of the pulse transit time (PTT). (**Mid Panel**) SCG signal with indication of the fiducial points associated with the Opening and Closing of the Aortic and Mitral valves (AO, AC, MO, and MC) to be considered for the estimation of the isovolumic contraction time (IVCT) and isovolumic relaxation time (IVRT), two clinical indexes of cardiac contractility and relaxation. (**Lower Panel**) PPG signal with indication of the timing for the PTT estimate.

The typical SCG waveform is illustrated in the middle panel of Figure 1. Further details on this signal and its derived parameters may be found in Reference [7].

Another signal frequently considered in the assessment of cardiovascular performance is the photoplethysmogram (PPG), namely, the measure of the light absorbed by the blood flowing into the arteries. An example of a PPG waveform is shown in the lower panel of Figure 1. This signal is commonly employed for estimating the blood oxygen saturation using red and infrared LED lights [12]. The PPG may also be exploited to track changes in the vessel diameter caused by the travelling of the blood pressure pulse. This information can be used to investigate features of the pressure pulse waveform and detect the pulse arrival at a given location of the vascular tree [13].

Even though ECG, SCG, and PPG individually provide rich information on cardiac and vascular performance, additional information may be derived when two or all three of these signals are simultaneously recorded and their reciprocal relationship investigated. For example, when PPG and ECG are concurrently recorded, the pulse transit time (PTT) can be measured. As schematized in the lower panel of Figure 1, this parameter is commonly estimated as the time delay from the R wave of the ECG and the arrival of the pressure pulse at a distal arterial site, usually the fingertip, earlobe or forehead (as detected by the PPG). The PTT may also be estimated by placing two PPG sensors on two different arterial sites and measuring the transit time of the pulse wave between sensors. The PTT inversely depends on the vascular stiffness, peripheral arterial resistance, and blood pressure, and its assessment provides us with integrated information on the vascular characteristics [14,15]. Further examples of simultaneous measures are illustrated in Section 3.2. At present, when concomitant recordings of multiple signals are needed, they are obtained by combining data from independent devices. The handling of multiple systems may lead to difficulties in the subject’s instrumentation, data collection, and time synchronization among signals.

As part of our research activity in the cardiovascular area, we recently activated a project requiring the simultaneous measure of the above three signals for the monitoring of healthy subjects and heart failure patients. For this purpose, a specific acquisition platform named SeisMote was developed. In the following, we describe the new system and its performances and illustrate examples of its current applications.

## 2. Methods

The SeisMote system consists of a wireless network of 12 sensorized nodes. The overall architecture was designed to (1) be wearable and unobtrusive during daily activities and sleep; (2) allow a possible simultaneous assessment of each signal from different body sites (by placing more nodes containing the same type of sensor on different body spots), (3) provide a time synchronization among different nodes with a maximal error of 1 ms; (4) guarantee at least 10 h of continuous recording; and (5) facilitate the possible future inclusion of additional types of sensors into the nodes.

In the development of the system, particular attention was paid to the efficiency of the data transmission so as to maintain low power consumption and guarantee the node connectivity with the proper time synchronization. As detailed in the following, none of the commercially available transmission protocols met all our needs and, thus, a custom protocol was developed.

**Figure 2:**
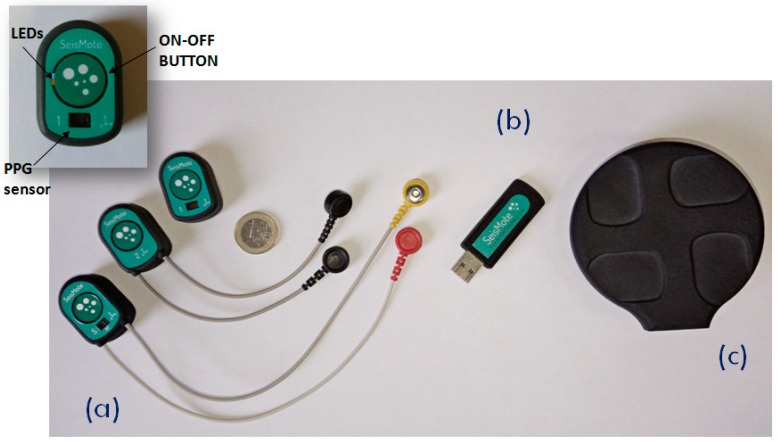
The hardware components of the SeisMote platform. (**a**) The sensorized nodes allowing the measure of different combinations of signals; from left to right: ECG–PPG–SCG, ECG–SCG, SCG–PPG. (**b**) The USB dongle (the hub). (**c**) The wireless recharger. Inset: A detail of a node and the position of the LEDs, switching button, and PPG sensor.

The system is composed of the wireless nodes, a USB dongle, which acts as network receiver, and the wireless battery recharger (see Figure 2), plus a software suit which includes a configuration/visualization program, a network file manager, and an Android app. Before each monitoring session the nodes are configured via software to select the signals to be acquired and one of the following monitoring modes:

- Real-Time mode (RT). In this mode, data are collected by multiple nodes and sent to the receiver (in the following “the hub”) which re-transmits them to a computer for a real-time visualization, analysis, and storage. In RT mode, the hub provides time synchronization to all nodes as detailed in the subsequent sections;
- The Off-Line mode (OL). In this mode, multiple nodes are used but data are locally stored on the memory card of each node;
- The Bluetooth Real-Time mode (BRT). A single node in the system may also be configured to allow connection to smartphones and tablets with the BluetoothLE (BLE) protocol. However, as hereafter detailed, BLE would not guarantee the proper time synchronization among different nodes and, thus, only one node at a time can be used in this mode.

### 2.1. The Hardware Architecture

#### 2.1.1. The Sensorized Node

Each node in the system has a size of 38 × 25 × 15 mm and weighs 10 grams.

**Figure 3:**
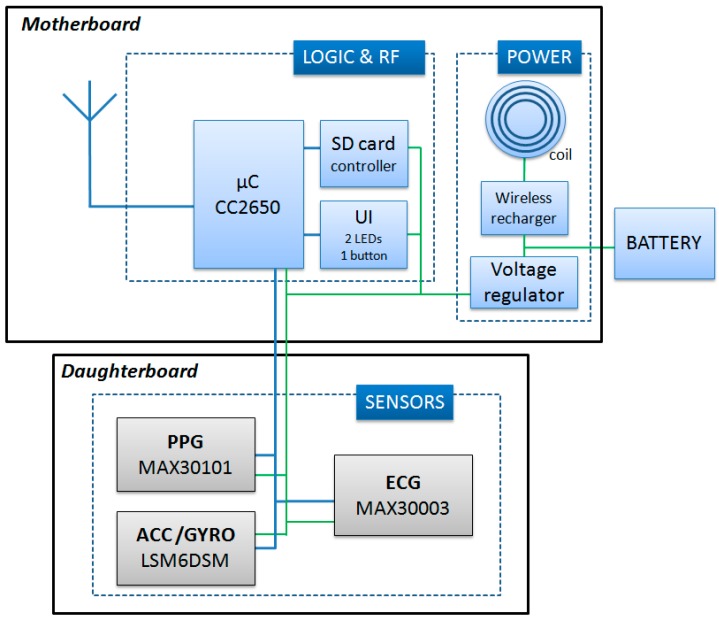
Scheme of the node circuit. UI = User interface; SD = Secure Digital.

As schematized in Figure 3, the node’s internal structure is composed of a motherboard and a daughterboard. The microcontroller (CC2650, Texas Instrument) is included in the motherboard. This component is based on the ARM-Cortex technology, has 8 kB of SRAM, 128 k of programmable flash memory, and an embedded 2.4 GHz RF transceiver. The motherboard also contains a secure digital memory card and the electronics for the wireless battery recharge and power supply.

The daughter board is 20 × 10 mm and is stacked on top of the motherboard. It contains a one-lead ECG front-end, a triaxial accelerometer (for the SCG measure), a triaxial gyroscope, and a green/red/infrared photoplethysmograph. Accelerations and rotational velocities are detected by the inertial unit LSM6DSM (ST microelectronics). For the SCG assessment, we need acceleration data with a resolution of 0.5 mg; the LSM6DSM component, when set with a full scale of ±2 g provides acceleration measures with a sensitivity of 0.061 mg/LSB, namely, with a resolution approximately ten times higher than needed by our applications. The PPG is detected by the MAX30101 (Maxim integrated) component, and the ECG analog front end is managed by the MAX30003 (Maxim integrated) chip which provides a clinical-grade signal. The node is powered by a polymer lithium-ion rechargeable battery with a capacity of 150 mAh. As shown in Figure 2a, nodes are available with various combinations of the above sensors. All signals are sampled at 200 Hz on 16 bits. Before transmission, sampled data may be encoded by the Adaptive Differential Pulse Code Modulation (ADPCM) algorithm [16] to improve the throughput of the network. The applicability and validity of this compression algorithm for the monitoring of biological signals has been previously tested [17,18].

#### 2.1.2. The Hub

When the system is functioning in the RT mode, a hub is needed to coordinate nodes and receive their data. In our platform, this role is played by a custom USB dongle (Figure 2b) containing the same CC2650 microcontroller with RF transceiver used in the node motherboard; the power supply is taken from the USB port. The dongle has the master role in the network; it regularly broadcasts a timestamp for the node synchronization, handles possible transmission errors, receives data from all nodes, creates a unique synchronized data stream, and send it to the PC via the USB port.

### 2.2. The Network Real-Time Mode

One challenging task of this project has been the development of the transmission protocol to be used when the system operates in the RT mode. Indeed, we needed a low-energy protocol able to connect up to 12 nodes with sufficient bandwidth and, importantly, capable to keep the time synchronization among nodes with a maximal error <1 ms. From a preliminary market survey, we soon discovered that none of commercially available low-power wireless technologies (e.g., ANT, ZigBee, Z-Wave, Bluetooth, BluetoothLE) fitted our requirements and, thus, we decided to design an ad-hoc protocol. Its details are provided in the following: Section 2.2.1, Section 2.2.2 and Section 2.2.3.

#### 2.2.1. Data Transmission

The unlicensed 2.4 GHz ISM (Industrial Scientific and Medical) band was used for the data transmission, and the star topology was adopted to transfer data from multiple slave nodes to the coordinator of the network, the hub, and vice versa.

To minimize the dimension of the nodes, it was decided that the transmission protocol had to be managed by the same microcontroller governing all the remaining node functions and not by a dedicated component. This policy required an optimization of CPU scheduling and memory resources to allow the concurrent running of the processes controlling data acquisition and data transmission.

**Figure 4:**
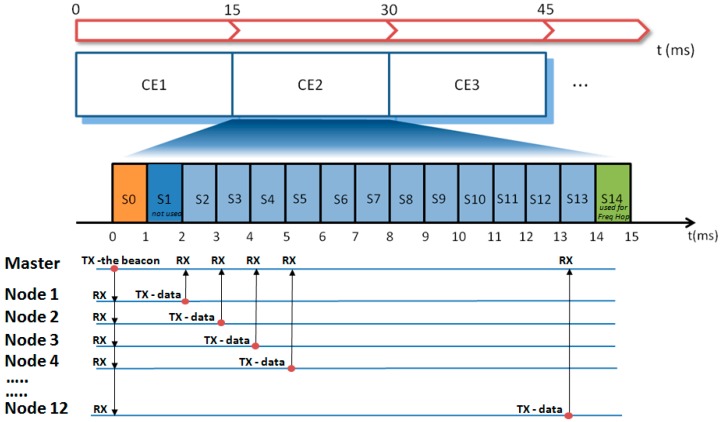
Diagram illustrating the TDMA (Time Domain Multiple Access) implementation and the data flow between the master (hub) and nodes at every connection event (CEn). Freq Hop = Frequency Hopping.

Coordination among nodes was achieved by the TDMA (Time Domain Multiple Access) methodology [19,20,21]. In TDMA, a certain RF channel is allocated for the access of one master and *N* slaves. In the channel, only one device at a time is allowed to transmit data; thus, different devices have dedicated time slots during which they can exclusively transmit. In our TDMA implementation, the transmission frame (in the following called “connection event” (CE)) is subdivided into 15 time slots, each lasting 1 ms (see Figure 4). The first slot, S0, is allocated to the master (the hub) to transmit a beacon packet to all nodes. This specific data packet contains a timestamp used for the time synchronization among nodes and possible additional network commands. Slots S2–S13 are allocated to the nodes 1–12 for data transmission. Slots S1 and S14 are not used. In particular, S1 is reserved to leave sufficient time to node 1 for the execution of possible commands received from the master before the data transmission in S2. Slot S14 is reserved to allow the actuation of the frequency hopping (see hereafter). When all 12 nodes are active in the network, each node has one slot assigned. In the case of fewer nodes, a single node may have up to 3 slots assigned for every CE to be used for the transmission of more signals or for allowing more time for data retransmission in case of error recovery.

Parenthetically, the adoption of the TDMA technique also leads to a reduction in power consumption. Indeed, as shown in Figure 4, during the beacon transmission, all slaves listen to the master, but in the subsequent phase, each node transmits data in the assigned time slot and remains in the idle state for the remaining time. This means that from a CE to the subsequent one, the RF subsystem of each node remains in the idle state for most of the time.

The 2.4 GHz ISM band is also used by WiFi, Bluetooth, and proprietary protocols. Thus, it is possible that other devices are working exactly in the same RF channel used by our platform; in this case, the transmitted data may be corrupted. To limit the negative effects of this eventuality, the transmission frequencies are changed over time through the frequency hopping technique [19,22,23] at every CE. Frequency hopping is also useful to counteract the effect of the multi-path fading, namely, the attenuation of the received RF power due to the multiple reflections of the electromagnetic waves [19]. Indeed, when transmitter and receiver do not operate in line-of-sight, the transmitted signal is reflected by walls or obstacles in the ambient. In this case, the signal can reach the receiver multiple times with different time delays due to the different propagation paths of the RF waves. In specific configurations of the obstacles, this phenomenon may result in a negative interference which reduces the power of the received signal. For any given ambient configuration, the extent of the power reduction depends on the frequency; thus, the possible fading effect can be mitigated over time by a regular change of the transmission channel.

#### 2.2.2. Data Fragmentation and Error Recovery

**Figure 5:**
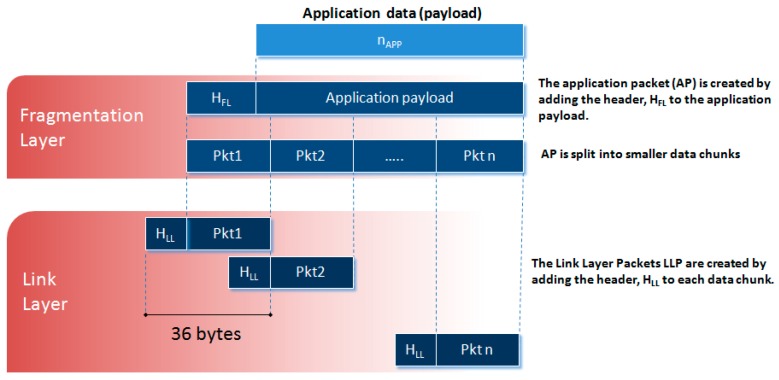
Diagram of the data fragmentation.

It is desired that the protocol be compatible with Application Packets of variable length also in view of possible future developments. For this purpose, a data fragmentation policy is adopted in our protocol (see Figure 5). It consists of the partitioning of the Application Packets into smaller fixed-length data chunks, the Link Layer Packets, which can be transmitted over multiple CEs. These chunks are then reassembled on the receiver side by a defragmentation procedure to reconstruct the original message. In the present implementation of the protocol, the Link Layer Packet has a size of 36 bytes.

Some transmitted packets may be lost in reception, however. This event may occur for different reasons: (1) because of the abovementioned fading effect; (2) because of the absorption phenomena of electromagnetic waves, e.g., due to the water content of the human body [24]; and (3) because of interference with other wireless devices using the same RF channel, notwithstanding the frequency hopping. A retransmission of the lost packets is implemented in the stack.

#### 2.2.3. Time Synchronization

In our applications, some cardiac parameters are computed as time delays among displacements occurring in signals collected by different nodes. Thus, we need that every node has the same vision of time with an error <1 ms. The quartzes of our nodes have a nominal frequency of 4 MHz with a tolerance of 10 ppm. This means that in the worst case and in absence of a time resynchronization, the accumulated time error between two nodes may reach 1 ms after only 50 s of transmission. This value may be reached considering only the possible intrinsic deviation in the nominal frequency of the quartzes. Additional changes in the quartz frequencies may also be expected from other factors including temperature. Therefore, the clock of every node in the network must be periodically adjusted to retain the same timekeeping. In the literature, there are three main types of synchronization within a network [25]: unidirectional, bidirectional, and reference broadcasting [26]. We preferred to keep the exchange of synch messages among the nodes at a minimal level; thus, we adopted the unidirectional synchronization procedure. By this method, *N* slaves (the nodes) are calibrated simultaneously through a single broadcast message sent by the master (the hub) which contains the timestamp. This message is embedded into the beacon. At every CE, each node receives the updated timestamp and adjusts the local clock.

### 2.3. The Network Off-Line Mode

When the system is in the OL mode, the hub is not used, and sensors’ data are locally stored in each node. At the end of the recording session data are downloaded from each node, pooled together and aligned over time by a custom program. This mode allows the data collection far from the receiver. If more than one node is used, during the initial configuration procedure, one of the nodes of the network is assigned the role of master to control the timekeeping. During the monitoring the master node transmits the beacon with the reference time to all the other nodes in the network every CE. The slave nodes receive the beacon and synchronize their own clock but do not transmit the sensor data to the master and rather store them on the local memory card. If only a single node is used, obviously no synch is required, and the wireless communication is disabled.

### 2.4. The BLE Real-Time Mode

This further functioning mode has been added to allow a real-time monitoring using a smartphone or tablet with a BLE connection. As already mentioned, the time synchronization among nodes cannot be guaranteed by the BLE protocol, and the throughput is limited; thus, only one node at a time can be used in this modality. In the designated nodes, the BLE stack has been included in the firmware replacing the code of the proprietary protocol.

**Figure 6:**
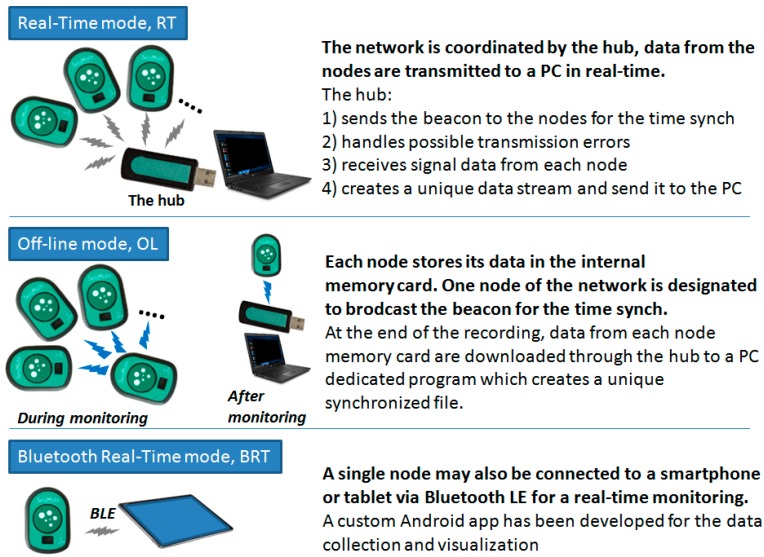
Scheme of the three monitoring modes.

A schematization of the three monitoring modes is illustrated in Figure 6.

## 3. Results

**Figure 7:**
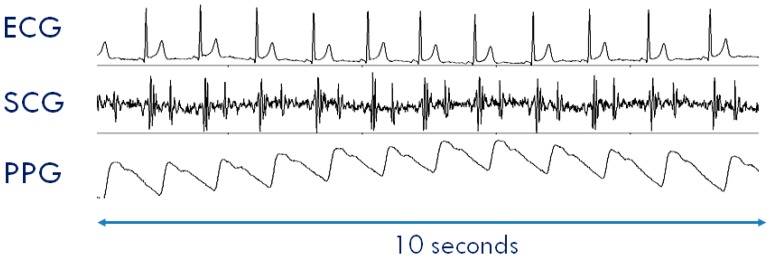
Example of ECG, SCG, and PPG collected by a SeisMote node. Data were collected in the setting illustrated in Figure 9.

An example of collected data is shown in Figure 7. Data were recorded in the setting illustrated in Figure 9.

### 3.1. System Performance

#### 3.1.1. Node Current Consumption

The total current consumption measured while the node was acquiring and transmitting data from all sensors was 9.4 mA which corresponded to approximately 16 h of continuous data monitoring for each battery recharge. The latter was obtained through a wireless charger in 2.5 h.

#### 3.1.2. Network Throughput

The bitrate of the radio chip was 500 kbps. As indicated in Section 2.2.1, at each CE, namely, every 15 ms, the node transmitted a Link Layer Packet (5 bytes for the header + 31 bytes of payload) which carried a chunk of application data. In a second, corresponding to 66.666 CEs, the throughput for each node was 31 × 66.666 = 2.07 kBps. For the whole network of 12 nodes, the total TP was 12 × 2.07 = 24.8 kBps = 198.7 kbps with an efficiency of 39.7%.

To estimate the TP, also in terms of the application data, it should be considered that in our protocol, the Application Packets have a header, *H_app_*, of 7 bytes independent from the length of the payload. Because of the splitting of the application packets into Link Layer Packets, in a single node, the link between application TP, *TP_app_*, and length of the application payload, *n_app_*, is described by the following formula:

*TP_app_*(*n_app_*) = *n_app_/round*((*n_app_* + *H_app_*)/*LLP*) × 66.666 where *LLP* is the length of the Link Layer payload (31 bytes), and the round function rounds the argument up to the nearest integer.

**Figure 8:**
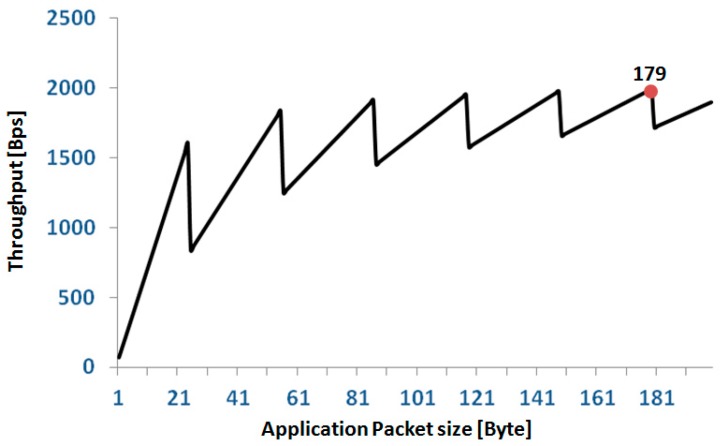
Relationship between application throughput and size of the application packet in a single node. The red spot indicates the application packet size selected for our protocol.

The relationship between *TP_app_* and *n_app_* has a sawtooth behavior as reported in Figure 8. We set the length of our application packets to 179 bytes; thus, from the above formula, the single node *TP_app_* is 1.99 kBps corresponding to a network *TP_app_* of 12 × 1.99 = 23.88 kBps = 190.9 kbps with an efficiency of 38.18%. For a full 12 node configuration, this setting allows each node to transmit up to three signals (16 bit @200 Hz). In the case of configurations with fewer nodes, the number of signals that can be transmitted progressively increases up to nine signals per node for a configuration of four nodes. In terms of error recovery, this setting allows a safe re-transmission of 6% of packets when all 36 signals are collected. Obviously, the allowed re-transmission rate increases if the number of collected signals is reduced.

We checked the quality of transmission in our laboratory. In all trials, we considered the full network configuration (i.e., 12 nodes), each transmitting three signals (we arbitrarily selected the *x*-, *y*- and *z*-axis of the acceleration); the nodes and receiver were in line-of-sight. Two types of tests were performed. In the first type (static), the 12 nodes were positioned close to each other on a tray, and the tray was located at 2, 5, 8, and 10 meters from the hub. Measures were taken for 5 min at each distance. The test was repeated three times. In the second group of tests (dynamic), measures were taken in a subject wearing the nodes and walking at 2, 5, 8, and 10 meters from the receiver for 3 min. Six nodes were placed on the front wall of the chest and the remaining six nodes on the back wall at the level of the 6th rib (just under the pectoral muscles). All nodes were in direct contact with the skin and were fastened by an elastic strap. Also, this test was repeated three times. During all recordings, WiFi was active in the area. For each test and each node we measured: (1) the received signal strength indicator (RSSI); (2) the percentage of Link Layer Packets retransmitted for the recovery of transmission errors; and (3) the number of Application Packets lost because of failure in the error recovery; lost application packets produce gaps inside the recording. Table 1 illustrates the results obtained from each test type. Values of RSSI and the percentage of retransmitted packets were averaged over the 12 nodes and the three test repetitions; the number of lost packets is the cumulative number of Application Packets lost by all nodes. It was apparent that 8 m is the maximal distance between subject and receiver for a good quality transmission.

#### 3.1.3. Time Synchronization

The jitter in the time synchronization among nodes was evaluated by measuring at every CE the discrepancy between the node local clock and the timestamp just transmitted by the master. In each node, the time was kept by a quartz-controlled counter advancing of a tick every 50 µs, thus our time measures were expressed in number of ticks. For the test, the node was programmed with a modified version of the firmware: the sensor signals were sampled (3 signals 16 bit @200 Hz) and the application packets prepared as usual, but at variance from the standard version, now these packets were not transmitted but rather trashed. Instead, a new packet with the difference in the timings was sent to the master. In this way, the processing load for the CPU was kept close to the real monitoring condition as much as possible. The measurement was taken with five different nodes, and each test lasted 20 min.

Results indicated that in every node the time discrepancy between consecutive resynchronization events ranged from 0 to 1 tick. These values correspond to a maximal jitter between nodes of 200 µs, i.e., well below the 1 ms threshold set in the project specification.

#### 3.1.4. Wearability

Each node in the system can be directly positioned on the body’s surface by adhesive tape, elastic straps, clips, or via integration into clothing. In our current application, where ECG and SCG were measured, the node/s were placed on the thorax. In this case, we usually applied a small piece of medical plaster to the chest and then attached the node to this substrate using a bi-adhesive tape. With this arrangement, the tape was not in direct contact with the skin and a strong adhesion tape may be used without risking possible skin irritations. This strategy was found to provide a comfortable and efficient bonding of the node to the body during movement and sweating. In addition, the small mass of the node makes it imperceptible while wearing, even during sleep.

**Figure 9:**
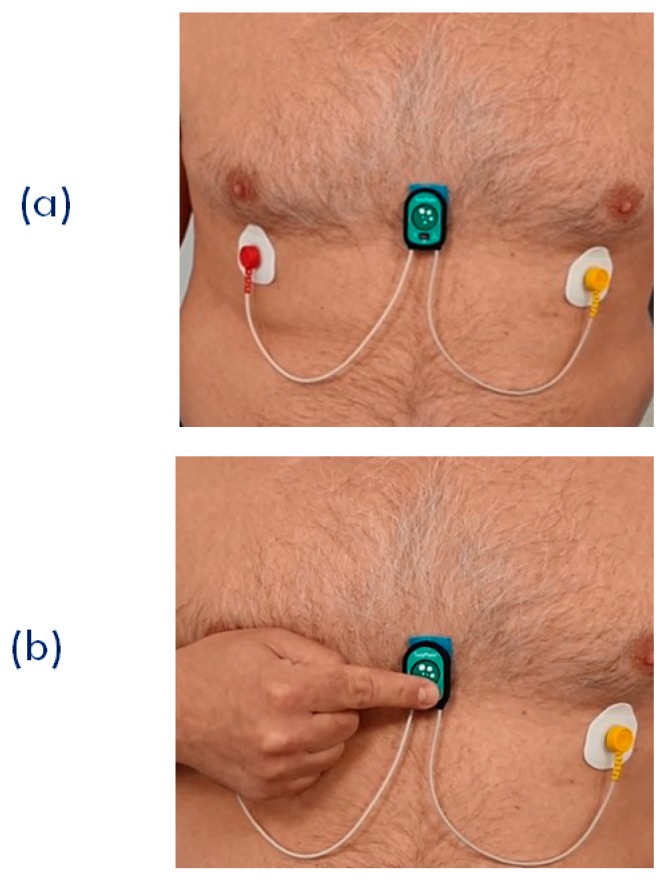
(**a**) Setting of the single node for the joint measurement of ECG, SCG, and PPG. This arrangement was used for the remote signal monitoring during the SideraB telerehabilitation program (see text). (**b**) Finger positioning for the PPG measurement.

**Figure 10:**
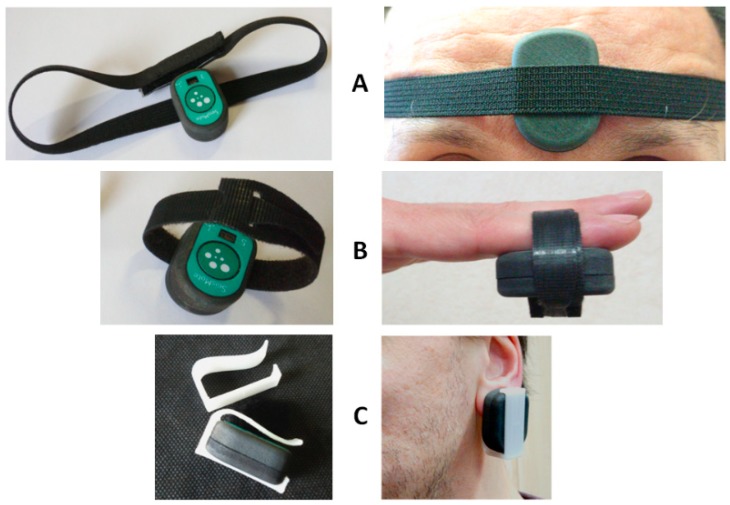
Adapters for the PPG measurement at the forehead (**A**), fingertip (**B**), and earlobe (**C**).

When the PPG is used, two different scenarios are possible. First, the subject stays still; a single node is used, and the node is placed on the sternum with the bi-adhesive tape for the ECG and SCG measurements (this arrangement is currently used for the monitoring of cardiac patients in the frame of the telerehabilitation project illustrated in the next section). In this case, PPG can be measured for short time periods by just putting the finger on the PPG sensor as shown in Figure 9. For a more general PPG measurement, we rotate the node so as to have the PPG sensor in contact with the skin, and specific adapters are used to keep the node adherent to the body. As shown in Figure 10, three types of adapters have been developed. The first is a clip for the PPG measure at the earlobe, and the second and the third are straps of different lengths that fasten the node to a finger and forehead, respectively.

### 3.2. Applications

**Figure 11:**
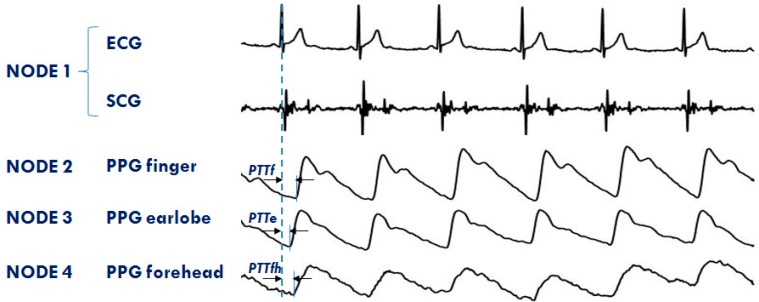
Example of signals from the 4 node configuration of the system aimed at investigating the PTT in different arterial sites: finger (PTTf), earlobe (PTTe), and forehead (PTTfh) by a multisite measurement of PPG.

The system is currently used in both the single- and multi-node configurations for the monitoring of healthy subjects and cardiac patients in a laboratory environment, telemedicine, and during sleep. The RT feature of the platform is now exploited to investigate the differences in the PTT dynamics when measured in various vascular districts simultaneously. Four nodes are used for this protocol. The first node is placed on the chest to measure ECG and SCG; the second, third, and forth node detect PPGs, respectively, at the fingertip, earlobe, and forehead. An example of a PPG multisite measurement is shown in Figure 11. The figure also illustrates how the PTTs are estimated from the ECG and PPG signals. Through the analysis of these data, we are now studying the strategies of local vascular blood pressure regulation.

**Figure 12:**
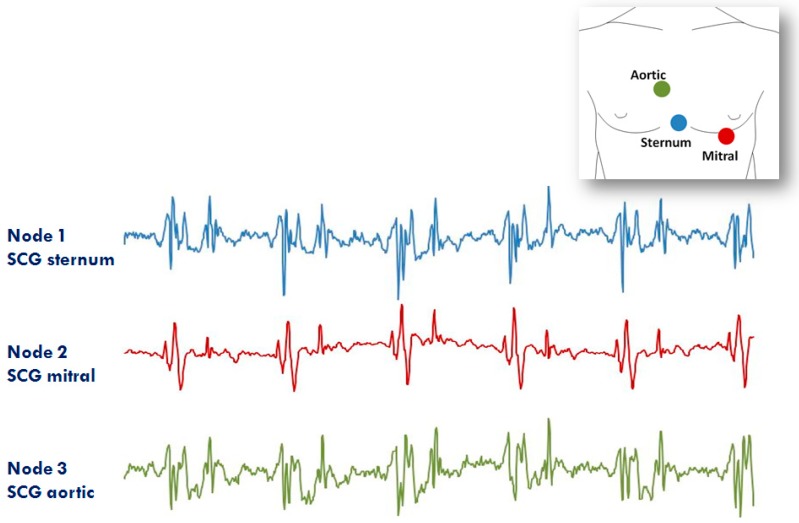
Example of signals from the 3 node configuration of the system aimed at investigating the SCG from different thorax sites (i.e., lower sternum, mitral, and aortic auscultation sites). Inset: positioning of the three nodes for the measure.

The second application of the system in the RT mode refers to the assessment of SCG from different precordial locations. The study was triggered by the observation that doctors auscultate the heart sounds from various chest sites to evaluate the different features of the heart’s performance. Similarly, it can be hypothesized that multisite measurement of SCG might provide more details on heart mechanics than a mono-site measurement. For this investigation, three nodes were used (additional nodes will be used in the future). They were placed on the lower part of the sternum (the traditional assessment site for SCG) in correspondence with the 2nd right intercostal space (the position for the aortic valve auscultation by the doctors) and on the heart apex (the position for the mitral valve auscultation). A diagram of the node position and an example of collected data are shown in Figure 12. It is apparent that, although common patterns are present in all signals, each individual SCG waveform is also characterized by peculiar features. We are now investigating the correlations between the SCG morphologies obtained from the multisite assessment and the real heart mechanical events visualized by ultrasound images.

Further applications of the system make use of single nodes. In the first ongoing study, the nodes are used to monitor ECG and SCG during sleep. The aim of the study is to investigate the correlation between heart rate variability and dynamic characteristics of the cardiac mechanics in this condition. For this protocol, the node is working in OL mode; thus, during monitoring, data are locally stored on the memory card. So far, 10 sleep recordings have been performed.

In the second application, single nodes were used in BRT mode to remotely monitor patients with heart failure during their telerehabilitation program at home. The study is part of a wide research project, SIDERA-B, financed by the Italian regional government of Lombardy, Regione Lombardia (POR FESR, id 232549), and aimed at testing new methodologies for the telerehabilitation of patients after hospital discharge. Each patient is guided by a tablet to do a series of physical activities and take a number of biomedical self-measurements every day. Also, once a week, a 3 min recording of ECG, SCG, and PPG from our device is taken. Signals are recorded by a single node placed on the sternum by adhesive tape as indicated in Section 3.1.4. while the patient is sitting. For the first two minutes, only the ECG and SCG are recorded, while, in the last minute, the patient places his/her finger on the PPG sensor (Figure 10) and also this signal is recorded. All devices, including our node, transmit data to the tablet via a BluetoothLE connection. Data are then re-transmitted to a central server that automatically prepares the reports and sends them to the cardiologists. The integration of the SeisMote node into the telerehabilitation platform, through the joint provision of ECG, indexes of cardiac mechanics and PTT, is intended to augment information on the patient’s health status. This should facilitate the evaluation of the effects of rehabilitation on the cardiovascular performance and a fast tuning of the exercise load on the basis of possible changes in the patient condition. This study is still in progress, and 48 recordings have so far been received and analyzed.

The above employment of the system is characterized by recordings without data gaps caused by transmission errors, and battery durations exceeding 16 h.

## 4. Discussion

In this article, a new wireless platform for the monitoring of cardiovascular performance was presented. The system was designed to guarantee flexibility of use in terms of the type of signals to be monitored, number of nodes, and functioning modalities.

SeisMote implements a wireless body area network (WBAN). Several platforms based on this paradigm have been proposed in the literature for the monitoring of vital signs (recent surveys may be found in References [27,28,29]). However, to the best of our knowledge, none of those platforms are characterized by the features we needed in terms of low-power consumption, number of nodes, and synch jitter. Two interesting solutions are commercially available, but one of them is based on the Bluetooth piconet thus limiting the number of connectable nodes to seven [30], and the other allows the connection to only three nodes [31]. None of the WBAN systems provides a SCG measure. Some of the systems currently available for SCG assessment may transmit sensor data to a receiver via a wireless connection [10,32,33], but they are essentially single nodes and are not part of a WBAN.

Thus, the SeisMote’s ability to measure up to 36 signals by dislocating 12 sensorized nodes in different parts of the body with an accuracy in the time synchronization better than 200 µs represents a unique feature. In particular, the latter characteristic allows a solid estimation of important biological parameters, such as PTT, based on the measure of time delays among signals collected by different nodes.

Another feature of the system is the possibility to have a multisite measure of the same signal. This aspect paves the way for interesting experimental applications. Two of them, namely, the multisite measure of accelerations and PPG (from which SCG and PTT were derived) have been described in Section 3.2. However, the possibility of obtaining simultaneous measures of single-lead ECGs by multiple nodes placed in different locations may also have practical relevance. Indeed, evidence is emerging that standard ECG leads, such as the Einthoven leads I, II, and III, might be synthesized by multiple single-lead ECG measurements [34].

Finally, a word on the layout of the node electronics. At present, the nodes include sensors for ECG, acceleration, gyroscope, and PPG measurement. However, their hardware architecture was designed to ease the integration of additional sensors by only the change of the daughterboard and keeping untouched the motherboard containing the microcontroller with the RF section, storage, and power supply electronics.

Future developments: In the current form, SeisMote allows the monitoring of signals, while the data analysis is performed offline. The next enhancement of the platform will include a DSP (Digital Dignal Processor) chip and more memory in the circuit of the node so as to provide also real-time computation of the derived parameters (such as the PTT and the indexes of cardiac mechanics). The second planned improvement will be an increase in the battery’s duration in order to allow monitoring over 24 h.

## Figures and Tables

**Table 1:** The results of the trial aimed at checking the quality of the transmission. RSSI = received signal strength indicator; %RP = percentage of retransmitted link layer packets.

**Static Tests**			
	*RSSI*	*%RP*	*Lost packets*
**2 m**	−55 dBm	0	0
**5 m**	−60 dBm	0	0
**8 m**	−63 dBm	0	0
**10 m**	−68 dBm	1.5%	0
**Dynamic Tests**			
**2 m**	−65 dBm	0	0
**5 m**	−73 dBm	2%	0
**8 m**	−78 dBm	5%	0
**10 m**	−83 dBm	9%	2
